# Supplementary material for: Comprehensive Chemical Profiling in the Ethanol Extract of Pluchea indica Aerial Parts by Liquid Chromatography/Mass Spectrometry Analysis of Its Silica Gel Column Chromatography Fractions
Source: Molecules. 2019 Jul 31;24(15):2784. doi: 10.3390/molecules24152784 (PMC6696005; doi:10.3390/molecules24152784)
Supplement: Supplementary file 1 [file molecules-24-02784-s001.pdf]

Supporting Information

# Comprehensive Chemical Profiling in the Ethanol Extract of Pluchea indica Aerial Parts by Liquid Chromatography/Mass Spectrometry Analysis of Its Silica Gel Column Chromatography Fractions

**JingYa Ruan [1], Jiejing Yan [2], Dandan Zheng [1], Fan Sun [2], Jianli Wang [2], Lifeng Han [2], Yi Zhang [1] and Tao Wang [1,2,*]**

[1] Tianjin State Key Laboratory of Modern Chinese Medicine, 312 Anshanxi Road, Nankai District, Tianjin 300193, China; Ruanjy19930919@163.com (J.R.); zhengdd1027@163.com (D.Z.); sf18435165322@163.com (F.S.)

[2] Tianjin Key Laboratory of TCM Chemistry and Analysis, Institute of Traditional Chinese Medicine, Tianjin University of Traditional Chinese Medicine, 312 Anshanxi Road, Nankai District, Tianjin 300193, China; 17320072093@163.com (J.Y.); wjl15802226160@126.com (J.W.); hanlifeng_1@sohu.com (L.H.)

* Correspondence: zhwwxzh@ tjutcm.edu.cn (Y.Z.); wangtao@tjutcm.edu.cn (T.W.); Tel./Fax: +86-22-5959-6168 (T.W.)

**Fig. S1.** The structures of 20 quinic acids standard references. ............................................ 5

**Fig. S2** The structures of 19 phenolic acids standard references. ........................................ 5

**Fig. S3**. The structures of 4 thiophenes standard references. ....................................... 6

**Fig. S4**. The structures of 13 flavonoids standard references. .......................................... 6

**Fig. S5**. The structures of 7 other compounds standard references. ................................... 6

**Fig. S6**. Base peak chromatograms (BPC) of PI on BEHC18, HSS C18, CSH FP, and T3 columns. ........................................................................................................ 7

**Fig. S7**. MS spectrum for peak 40.13 min from TIC of PI. ........................................... 7

**Fig. S8.** MS spectrum for peak 40.13 min from TIC of PIE4. ....................................... 7

**Fig. S9**. The EIC of *m*/*z* 677.15119. A: EIC from PI; B: EIC from PIE6. ................................ 8

**Fig. S10**. MS/MS spectrum of the [M−H]$^-$ ions for **11**, **16**, and **4**. ....................................... 8

**Fig. S11.** MS/MS spectrum of the [M−H]$^-$ ions for **3**, **5**, and **9**. ........................................... 9

**Fig. S12.** MS/MS spectrum of the [M−H]$^-$ ions for **18** and **20**. ......................................... 10

**Fig. S13.** MS/MS spectrum of the [M−H]$^-$ ions for **21**, **22**, **24**, **28** and **29**. ......................... 11

**Fig. S14.** The MS/MS spectrum of the [M−H]$^-$ ions for **35**, **37–40**, **45**. ................................ 12

**Fig. S15.** The MS/MS spectrum of the [M−H]$^-$ ions for peaks **46**, **48**, **49**, **50**, **51**, **55** and **61**. . 13

**Fig. S16.** The MS/MS spectrum of the [M−H]$^-$ ions for peaks **62**, **66** and **73**. ............................ 14

**Fig. S17.** The MS/MS spectrum of the [M−H]$^-$ ions for peaks **47**, **53**, **63** and **65**. ....................... 15

**Fig. S18.** The MS/MS spectrum of the [M−H]$^-$ ions for peaks **76** and **92**. ................................. 15

**Fig. S19.** The MS/MS spectrum of the [M−H]$^-$ ions for peaks **80**, **91** and **98**. ............................ 16

**Fig. S20.** The MS/MS spectrum of the [M−H]$^-$ ions for **1**, **7** and **12**. .......................................... 17

**Fig. S21.** The MS/MS spectrum of the [M−H]$^-$ ions for **8**, **17**. ................................................... 17

**Fig. S22.** The UPLC-MS analysis results of **67** standard references in negative and positive mode............................................................................................................................ 18

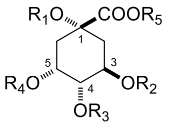

| | $R_1$ | $R_2$ | $R_3$ | $R_4$ | $R_5$ |
|---|---|---|---|---|---|
| **4:** | H | H | H | Caffeoyl | H |
| **11:** | H | Caffeoyl | H | H | H |
| **16:** | H | H | Caffeoyl | H | H |
| **29:** | H | Caffeoyl | H | H | $CH_3$ |
| **35:** | Caffeoyl | H | Caffeoyl | H | H |
| **37:** | Caffeoyl | H | H | Caffeoyl | H |
| **38:** | Caffeoyl | Caffeoyl | H | H | H |
| **39:** | H | H | Caffeoyl | Caffeoyl | H |
| **40:** | H | Caffeoyl | H | Caffeoyl | H |
| **45:** | H | Caffeoyl | Caffeoyl | H | H |
| **47:** | Caffeoyl | Caffeoyl | H | Caffeoyl | H |
| **51:** | H | H | Caffeoyl | Caffeoyl | $CH_3$ |
| **53:** | Caffeoyl | Caffeoyl | Caffeoyl | H | H |
| **55:** | H | Caffeoyl | H | Caffeoyl | $CH_3$ |
| **61:** | H | Caffeoyl | Caffeoyl | H | $CH_3$ |
| **65:** | H | Caffeoyl | Caffeoyl | Caffeoyl | H |
| **66:** | H | Caffeoyl | H | Caffeoyl | $CH_2CH_3$ |
| **74:** | H | Caffeoyl | Caffeoyl | H | $CH_2CH_3$ |
| **76:** | Caffeoyl | Caffeoyl | Caffeoyl | Caffeoyl | H |
| **91:** | H | Caffeoyl | Caffeoyl | Caffeoyl | $CH_3$ |

3,4,5-Trihydroxycinnamoyl: Caffeoyl: Coumaroyl: Ferulyl:

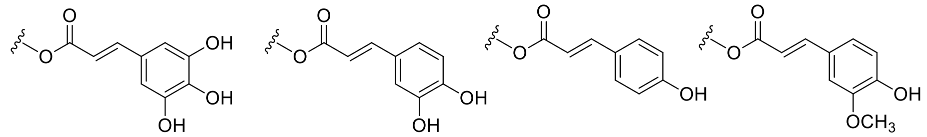

**Fig. S1.** The structures of 20 quinic acids standard references.

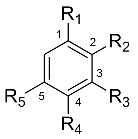

| | $R_1$ | $R_2$ | $R_3$ | $R_4$ | $R_5$ |
|---|---|---|---|---|---|
| **1:** | COOH | H | OH | OH | H |
| **6:** | COOH | H | H | OH | H |
| **7:** | CHO | H | OH | OH | H |
| **10:** | CHO | H | $OCH_3$ | OH | OH |
| **12:** | COOH | H | $OCH_3$ | OH | H |
| **19:** | CHO | H | $OCH_3$ | OH | H |
| **23:** | CHO | H | $OCH_3$ | OH | $OCH_3$ |
| **109:** | $COOC_4H_9$ | $COOC_4H_9$ | H | H | H |

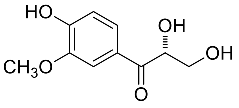

**5**

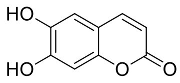

**13**

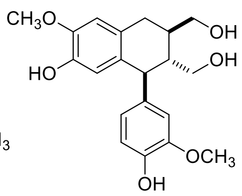

**33**

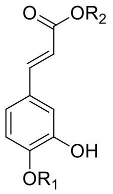

| | $R_1$ | $R_2$ |
|---|---|---|
| **14:** | H | H |
| **34:** | $CH_3$ | H |
| **52:** | H | $C_2H_5$ |

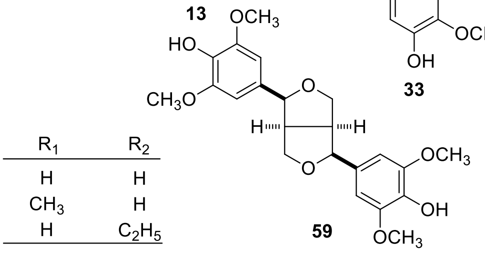

**59**

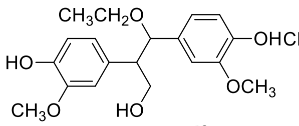

**73**

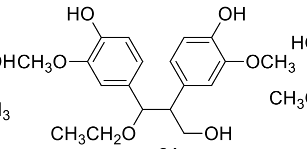

**81**

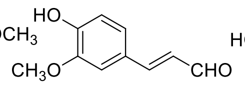

**112**

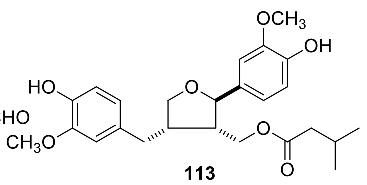

**113**

**Fig. S2.** The structures of 19 phenolic acids standard references.

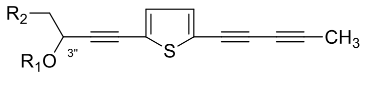

| | $R_1$ | $R_2$ | 3'' |
|---|---|---|---|
| **99:** | H | OH | *R* |
| **105:** | H | $OCCH_3$ | *R* |
| **107:** | $CH_2CH_3$ | OH | *S* |
| **111:** | $CH_2CH_3$ | $OCCH_3$ | *S* |

**Fig. S3.** The structures of 4 thiophenes standard references.

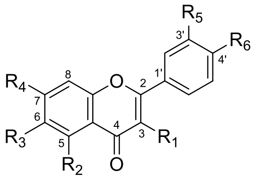

| | $R_1$ | $R_2$ | $R_3$ | $R_4$ | $R_5$ | $R_6$ |
|---|---|---|---|---|---|---|
| **31:** | OGa | OH | H | OH | OH | OH |
| **32:** | OGlc | OH | H | OH | OH | OH |
| **42:** | OGlc | OH | H | OH | H | OH |
| **43:** | H | OH | H | OGlc | OH | OH |
| **44:** | $OCH_3$ | OH | H | OH | OGlc | OH |
| **54:** | OH | OH | H | OH | OH | OH |
| **57:** | H | OH | H | OH | OH | OH |
| **68:** | $OCH_3$ | OH | OH | $OCH_3$ | H | OH |
| **71:** | OH | OH | H | OH | H | OH |
| **77:** | OH | OH | H | OH | $OCH_3$ | OH |
| **86:** | $OCH_3$ | OH | OH | $OCH_3$ | $OCH_3$ | OH |
| **87:** | $OCH_3$ | OH | $OCH_3$ | OH | OH | $OCH_3$ |
| **101:** | $OCH_3$ | OH | $OCH_3$ | $OCH_3$ | OH | $OCH_3$ |

Glc: $\beta$-D-glucopyranoside; Gal: $\beta$-D-galactopyranoside

**Fig. S4.** The structures of 13 flavonoids standard references.

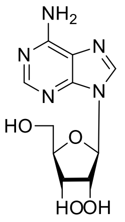

**2**

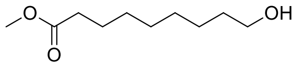

**64**

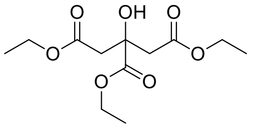

**56**

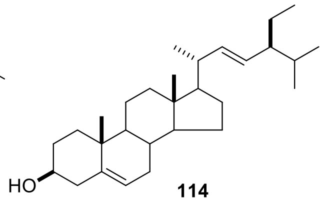

**114**

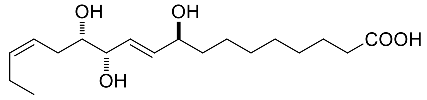

**75**

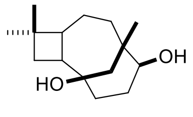

**85**

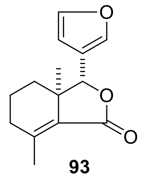

**93**

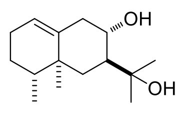

**94**

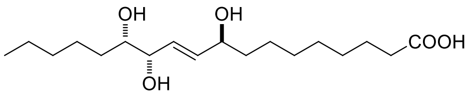

**83**

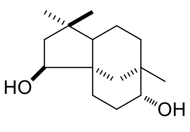

**100**

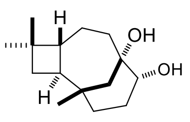

**102**

**Fig. S5.** The structures of 7 other compounds standard references.

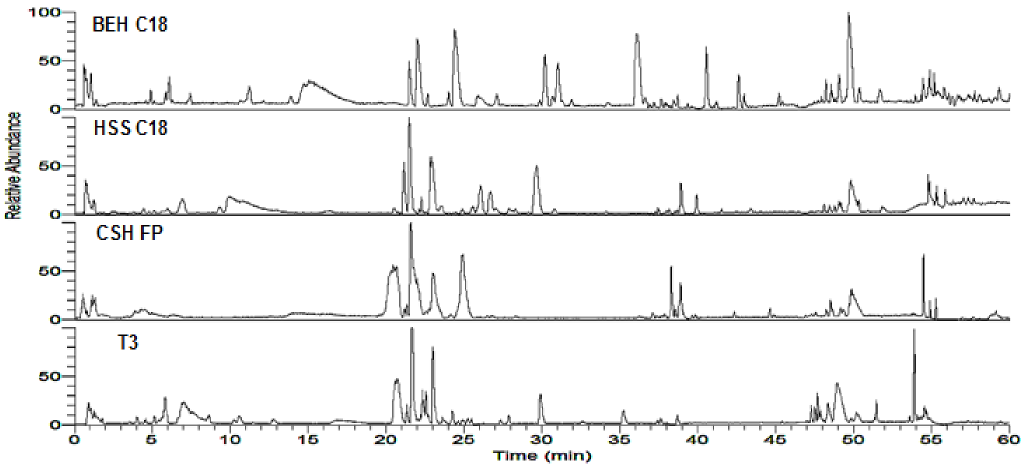


**Fig. S6.** Base peak chromatograms (BPC) of PI on BEHC18, HSS C18, CSH FP, and T3 columns.

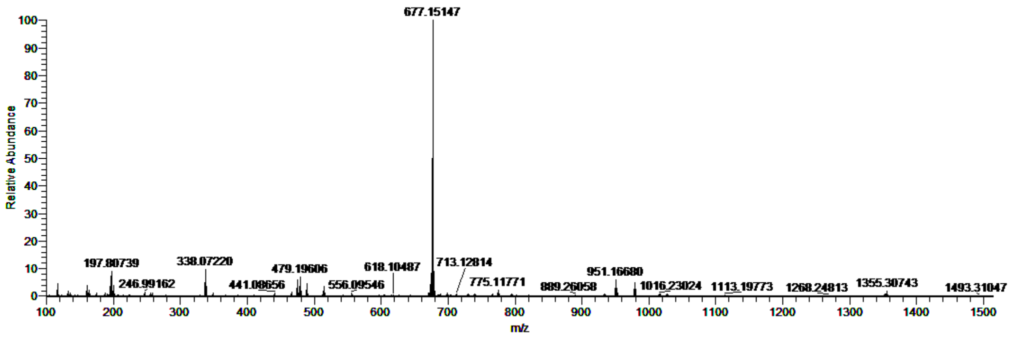


**Fig. S7**. MS spectrum for peak 40.13 min from TIC of PI.

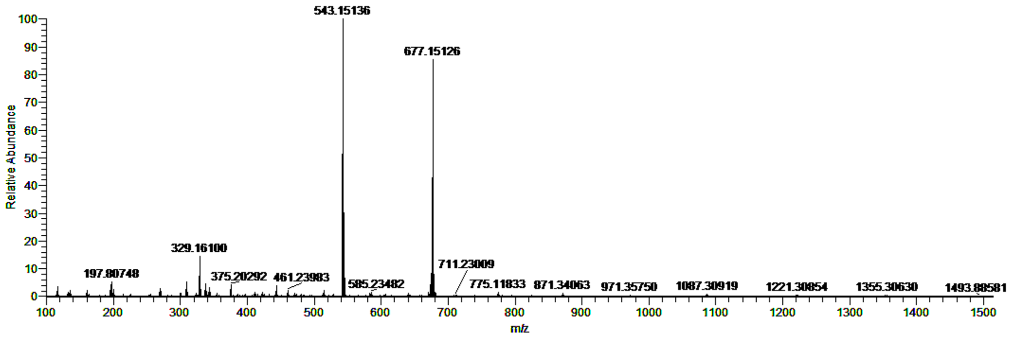


**Fig. S8**. MS spectrum for peak 40.13 min from TIC of PIE4.

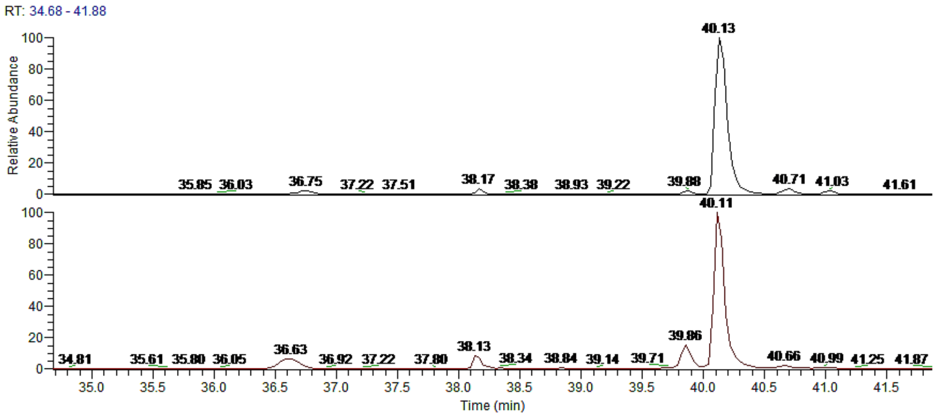


**Fig. S9**. The EIC of *m*/*z* 677.15119. A: EIC from PI; B: EIC from PIE6.

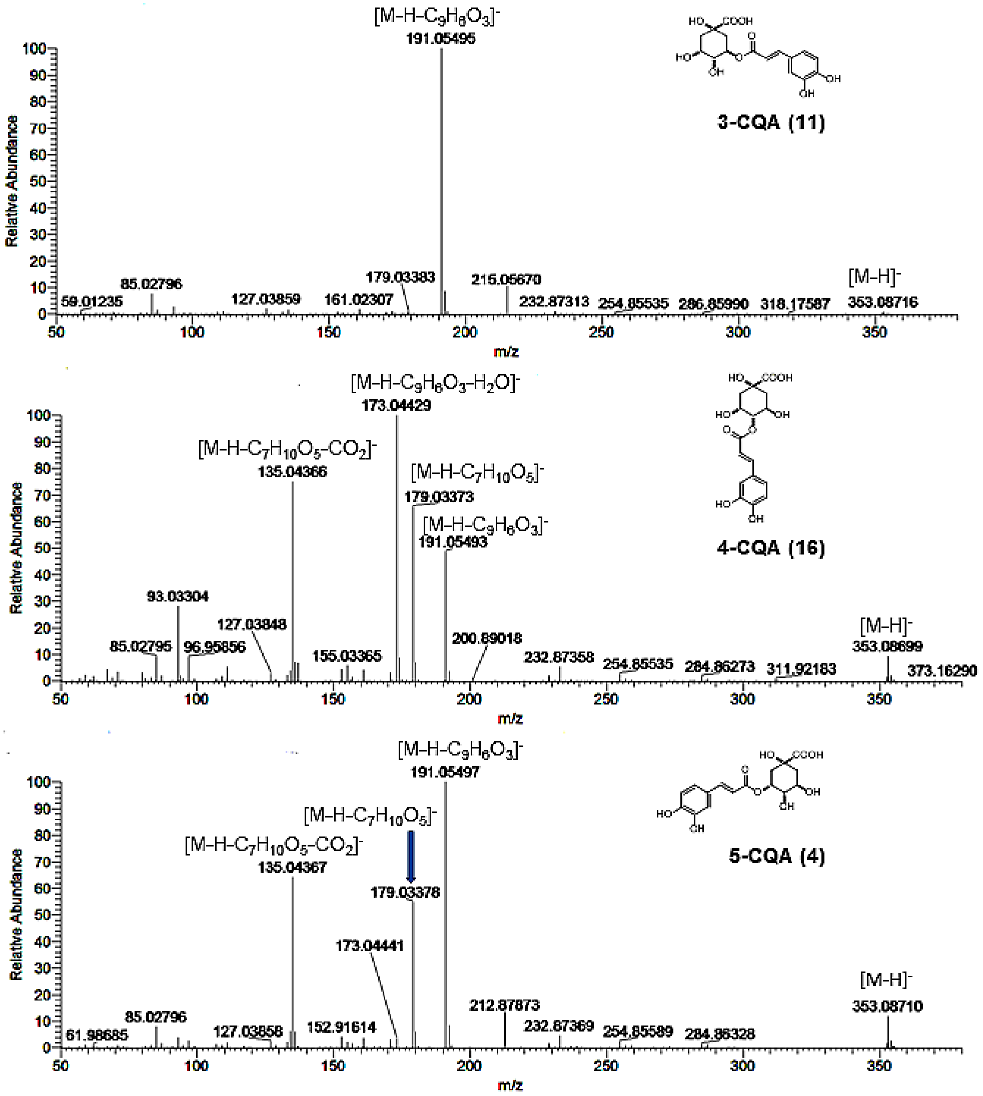


**Fig. S10**. MS/MS spectrum of the [M−H]⁻ ions for **11**, **16**, and **4**.

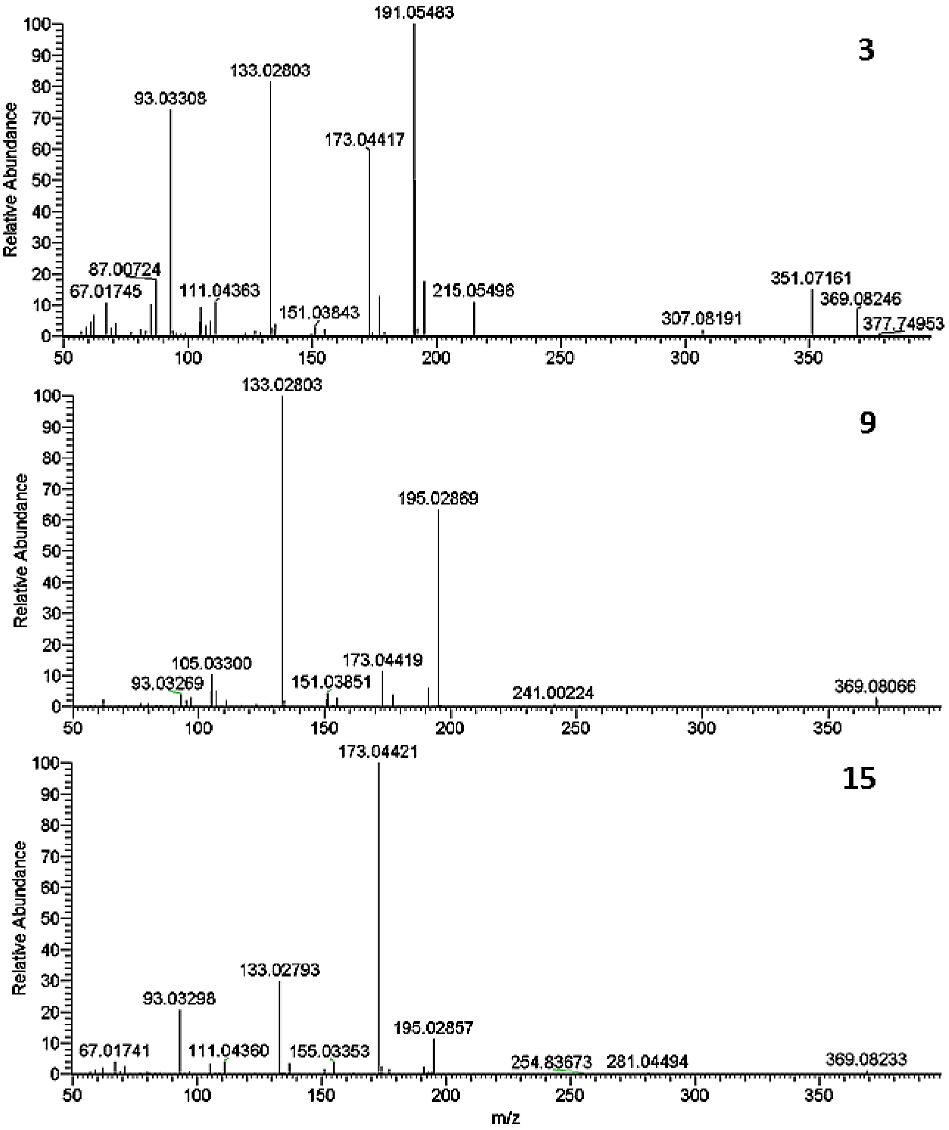


**Fig. S11**. MS/MS spectrum of the [M−H]$^-$ ions for **3**, **5**, and **9**.

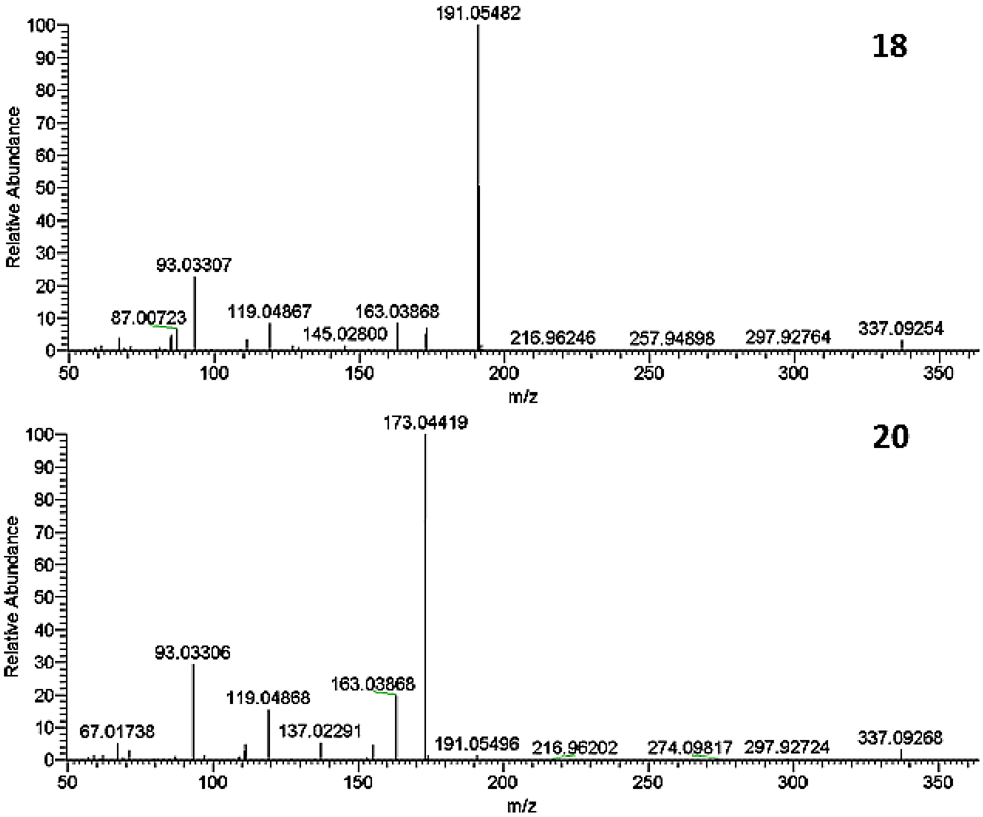


**Fig. S12**. MS/MS spectrum of the [M−H]⁻ ions for **18** and **20**.

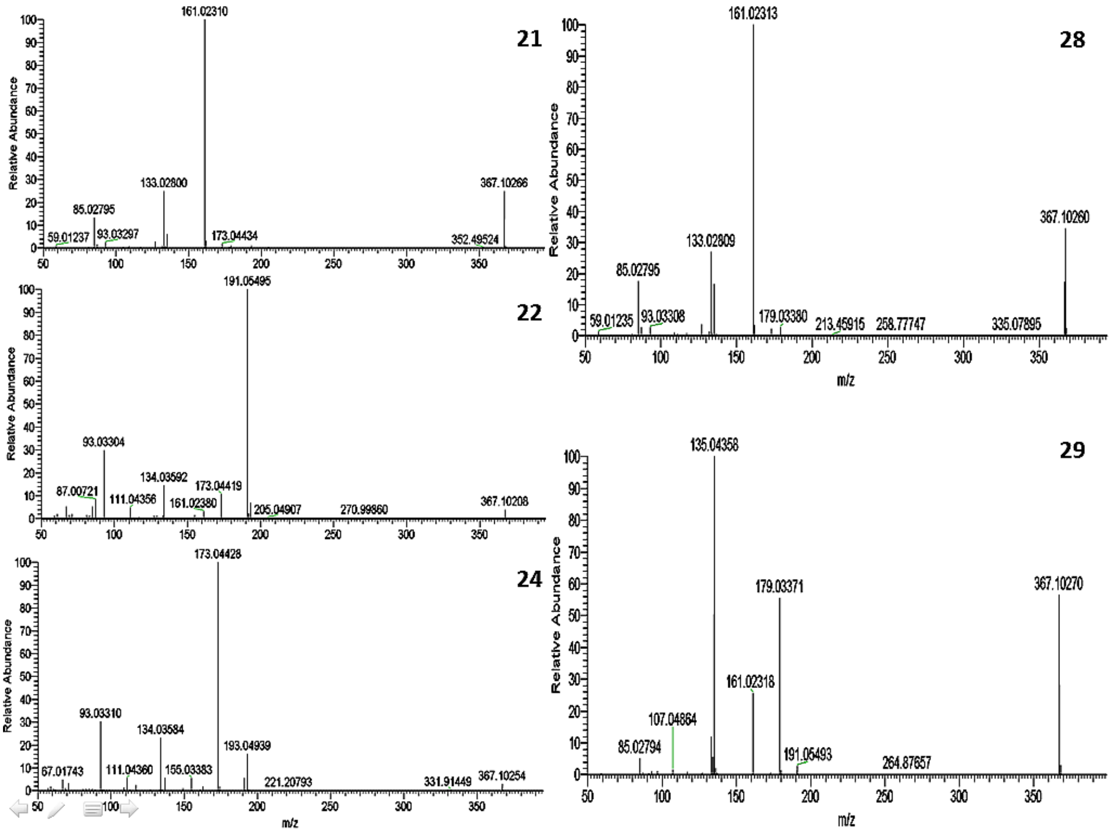


**Fig. S13**. MS/MS spectrum of the [M−H]⁻ ions for **21**, **22**, **24**, **28** and **29**.

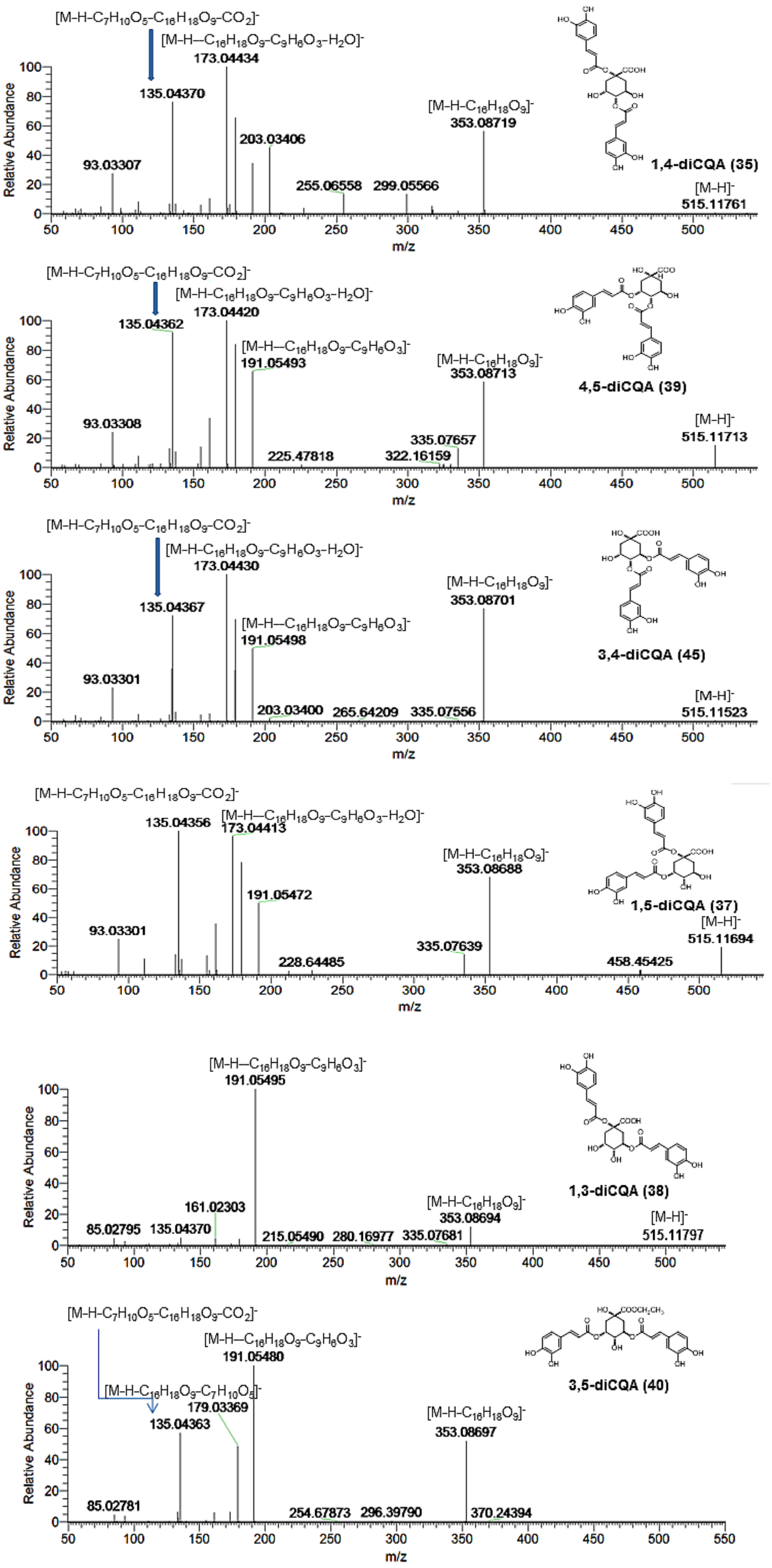


**Fig. S14**. The MS/MS spectrum of the [M−H]⁻ ions for **35**, **37**–**40**, **45**.

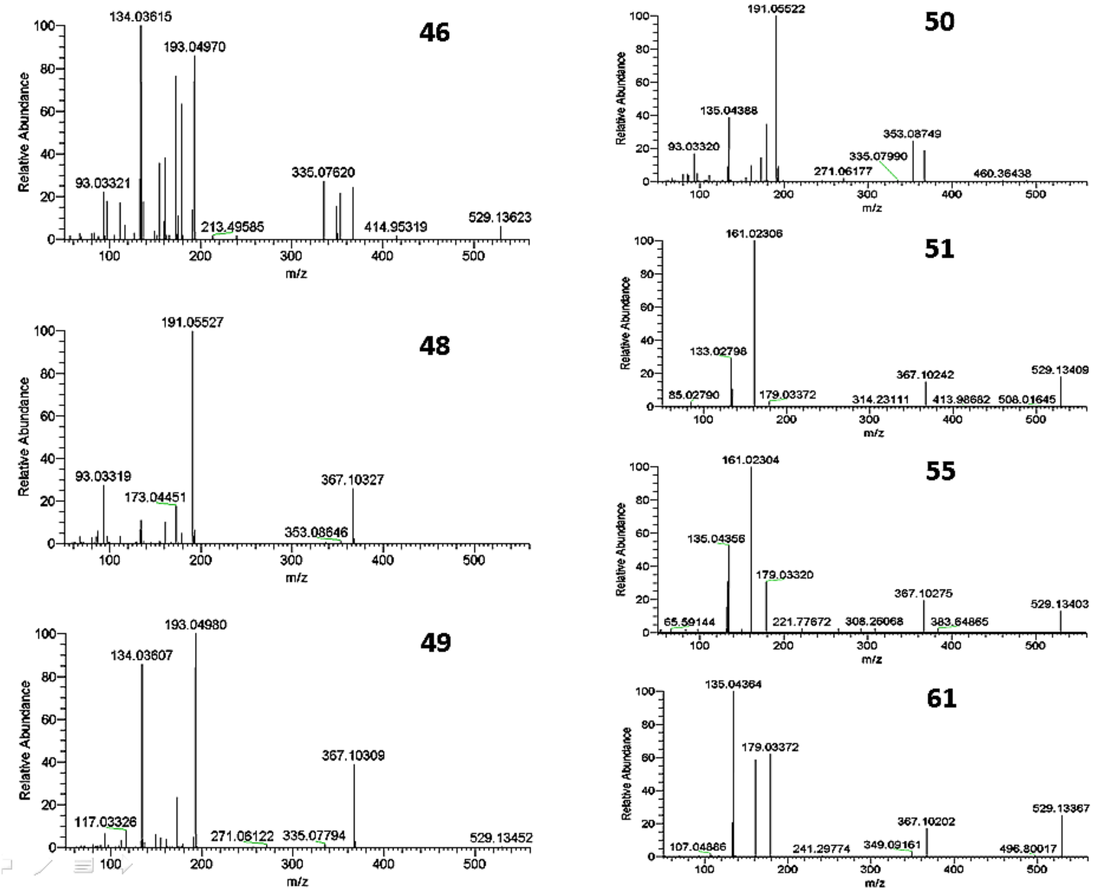


**Fig. S15.** The MS/MS spectrum of the [M−H]⁻ ions for peaks **46**, **48**, **49**, **50**, **51**, **55** and **61**.

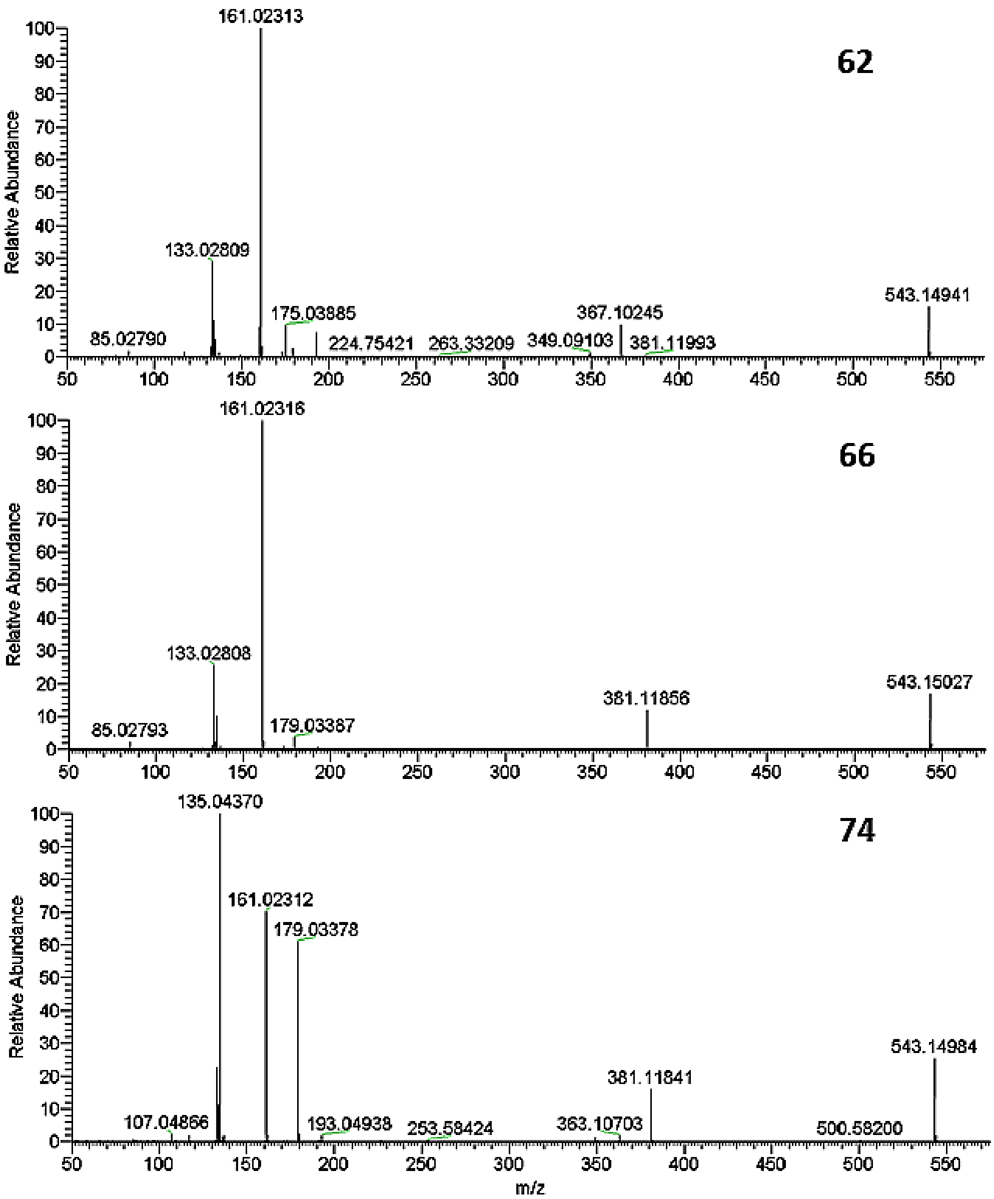


**Fig. S16.** The MS/MS spectrum of the [M−H]⁻ ions for peaks **62**, **66** and **73**.

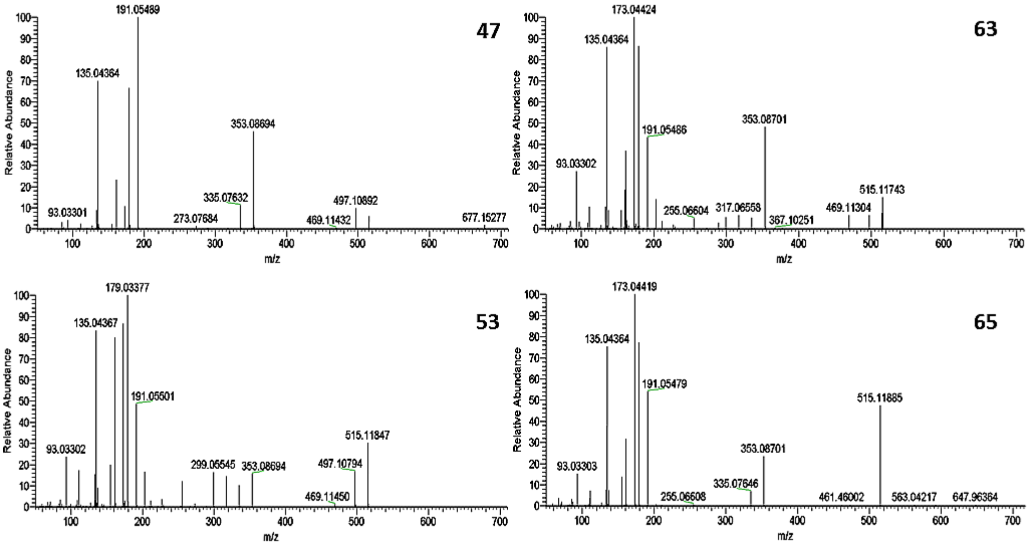


**Fig. S17.** The MS/MS spectrum of the [M−H]⁻ ions for peaks **47**, **53**, **63** and **65**.

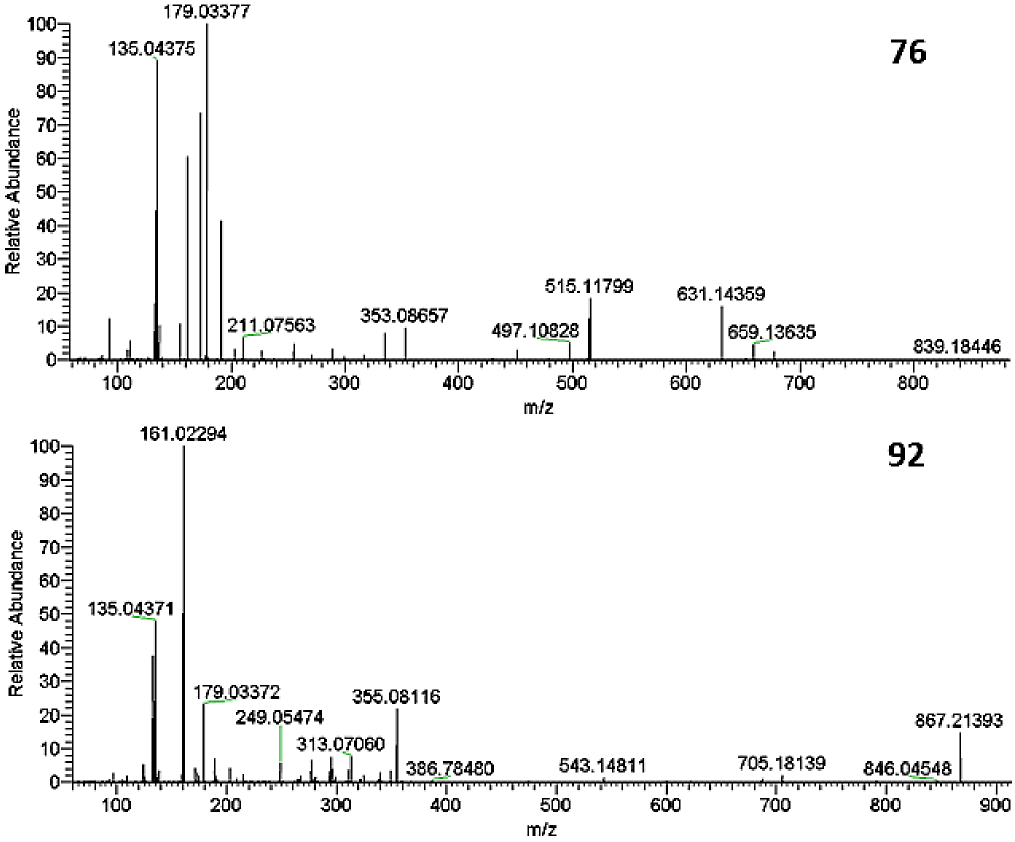


**Fig. S18.** The MS/MS spectrum of the [M−H]⁻ ions for peaks **76** and **92**.

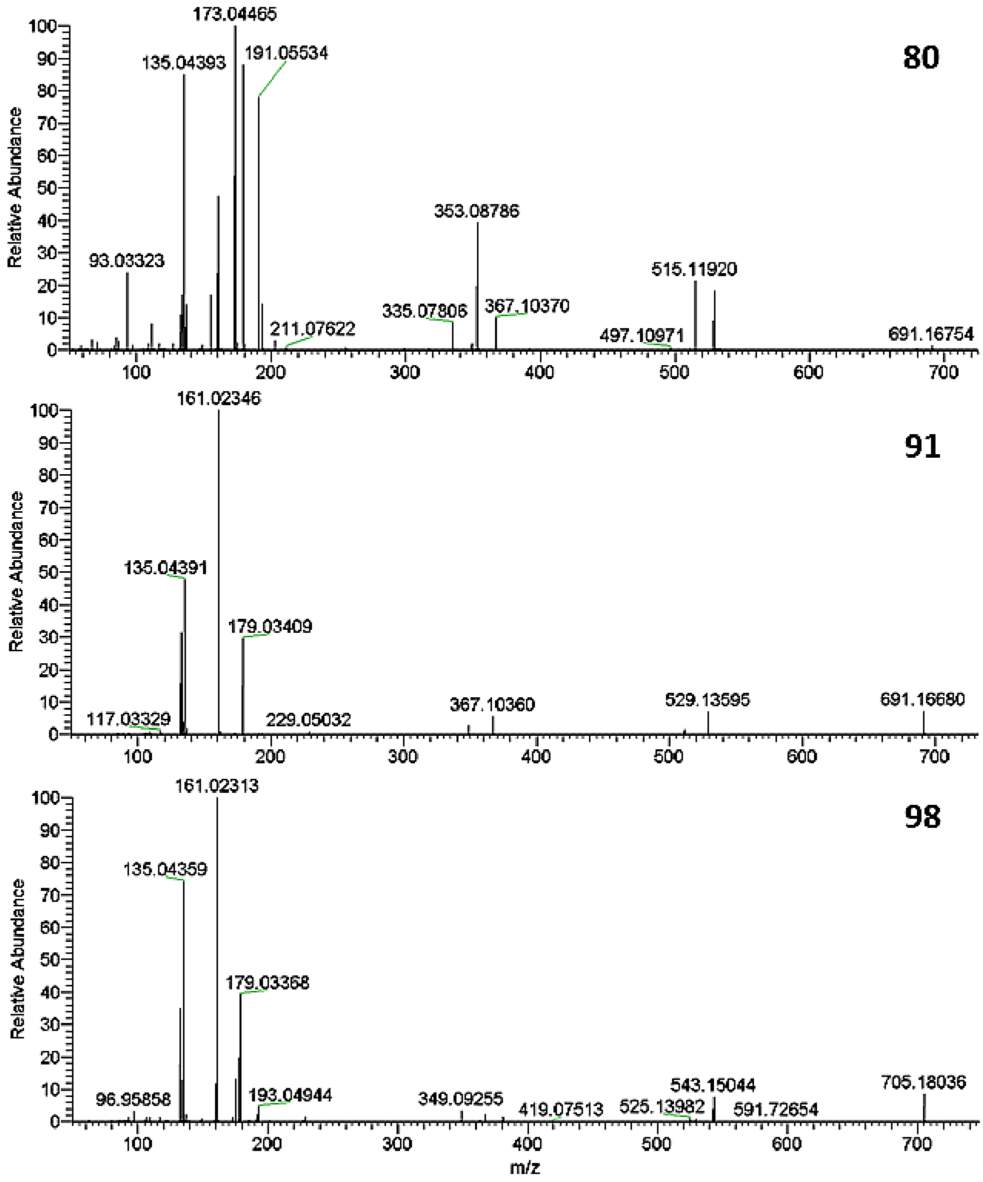


**Fig. S19.** The MS/MS spectrum of the [M−H]⁻ ions for peaks **80**, **91** and **98**.

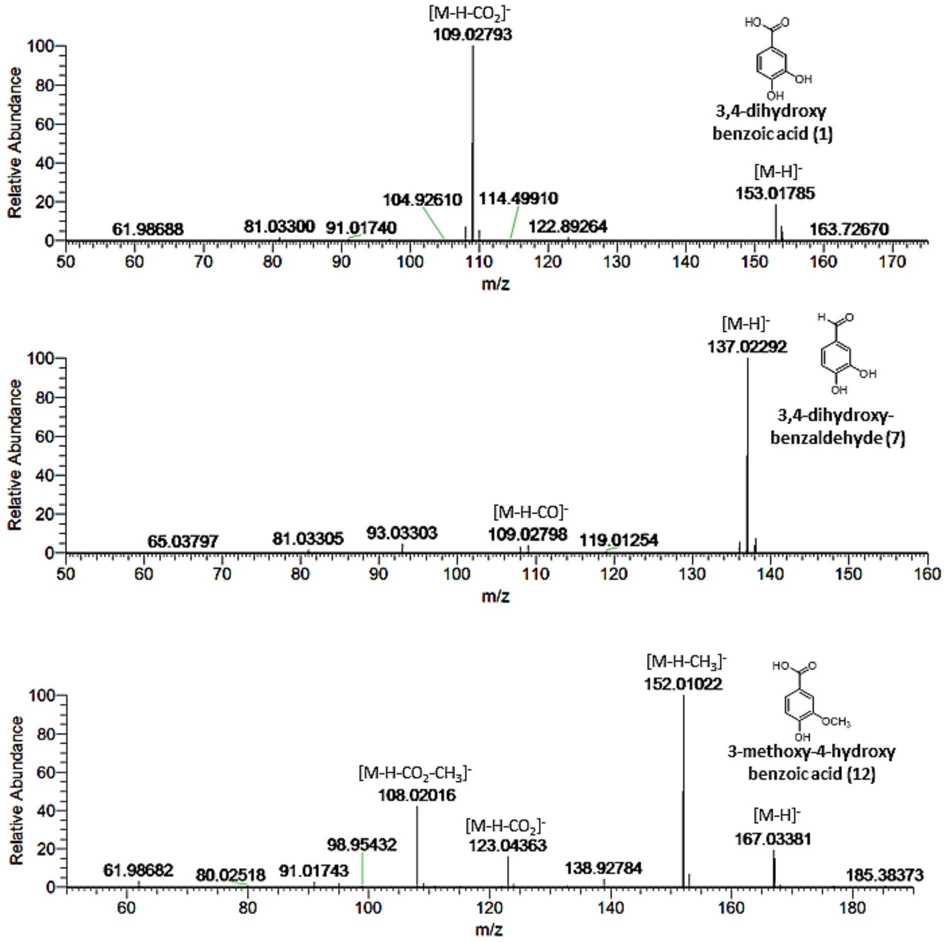


**Fig. S20.** The MS/MS spectrum of the [M−H]⁻ ions for **1**, **7** and **12**.

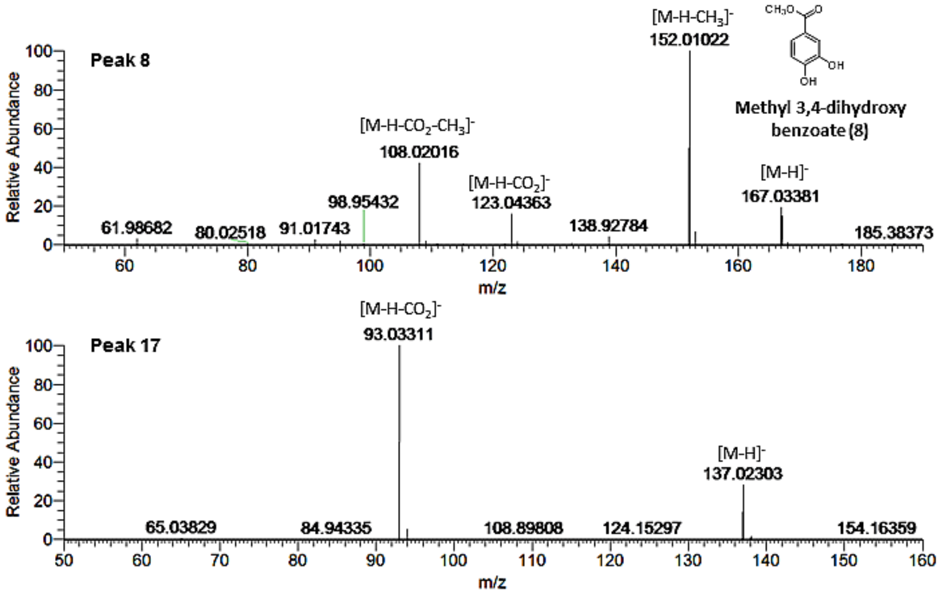


**Fig. S21.** The MS/MS spectrum of the [M−H]⁻ ions for **8**, **17**.

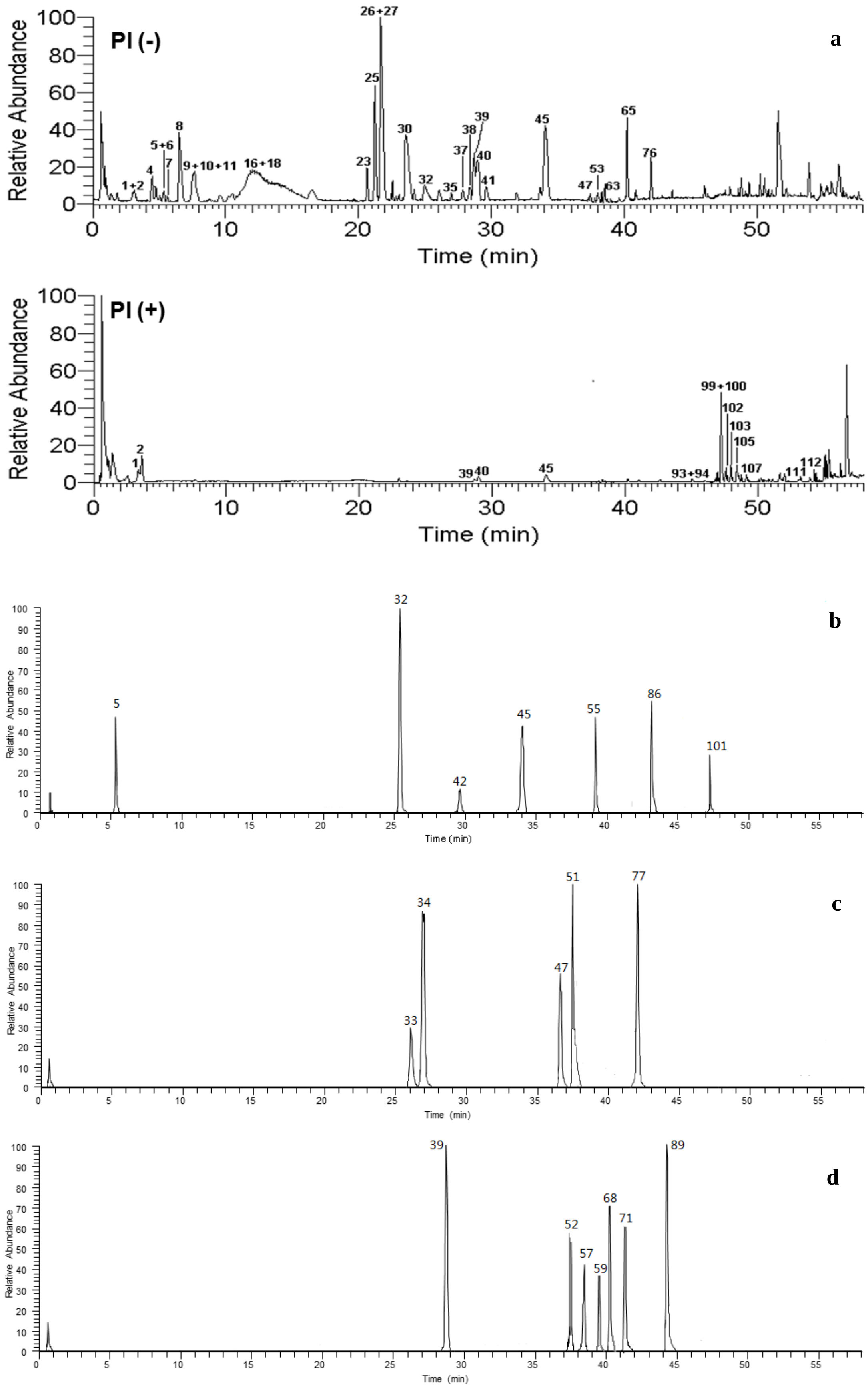
a
PI (-)
Relative Abundance
Time (min)
1+2
4
5+6
7
8
9+10+11
16+18
23
25
26+27
30
32
35
37
38
39
40
41
45
47
53
63
65
76
PI (+)
1
2
39
40
45
93+94
99+100
102
103
105
107
111
112
b
5
32
42
45
55
86
101
c
33
34
47
51
77
d
39
52
57
59
68
71
89

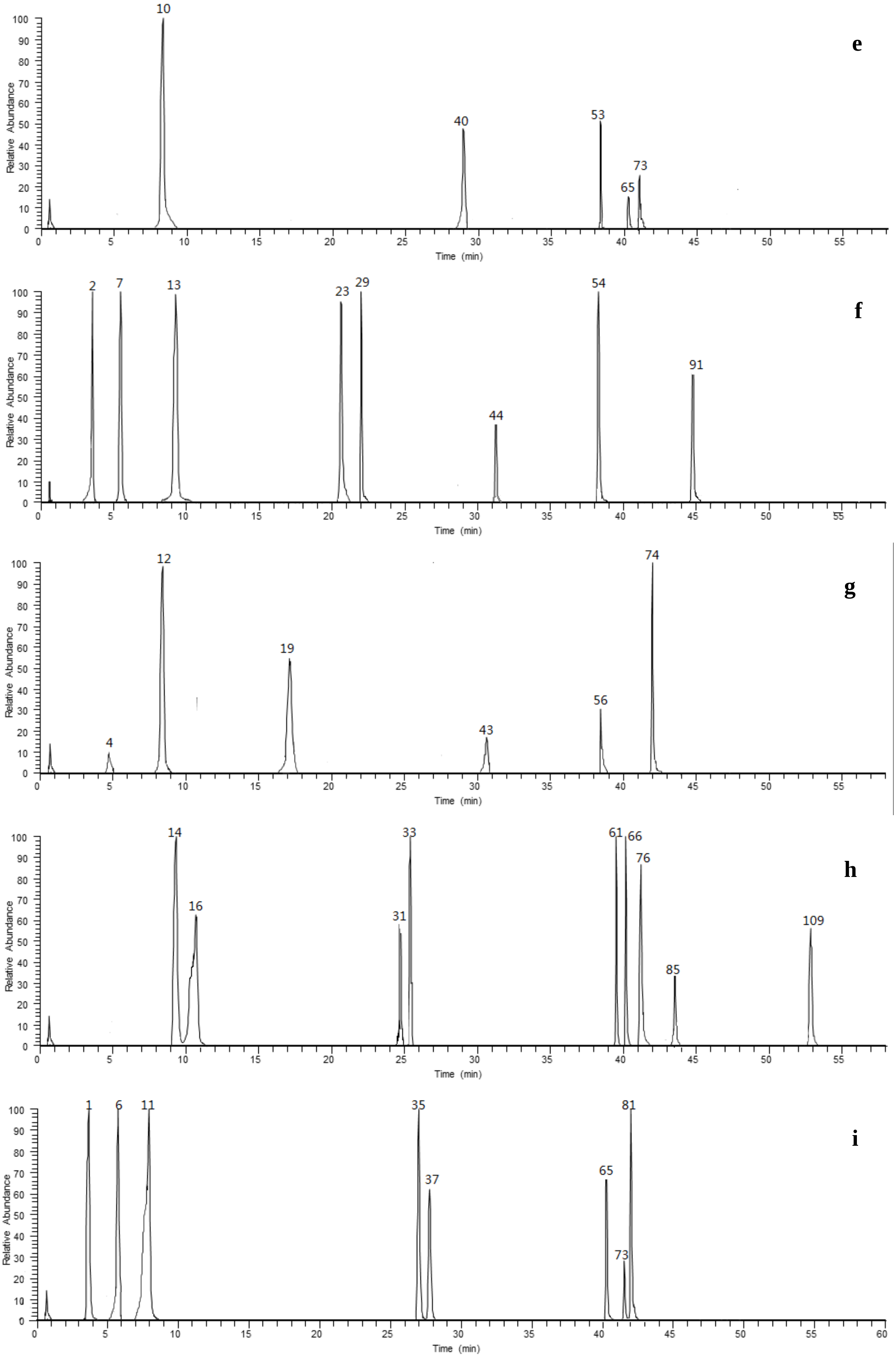
e
10
40
53
65
73
f
2
7
13
23
29
44
54
91
g
4
12
19
43
56
74
h
14
16
31
33
61
66
76
85
109
i
1
6
11
35
37
65
73
81
Relative Abundance
Time (min)

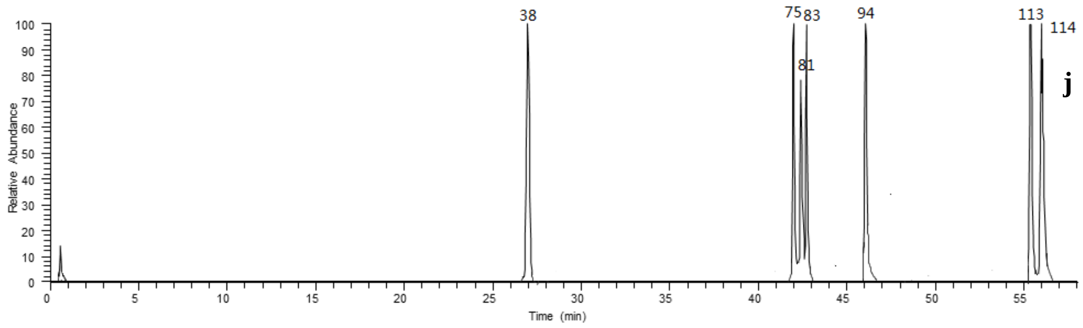


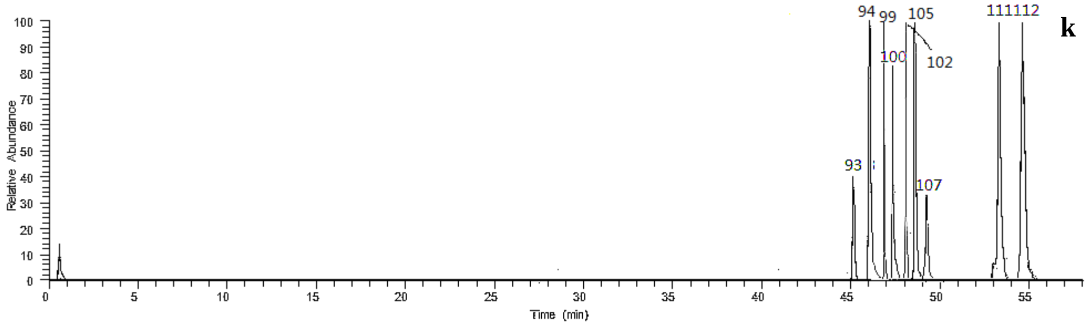


**Fig. S22.** The UPLC-MS analysis results of **67** standard references in negative and positive mode a: Base peak chromatograms of *P. indica* 70% EtOH extract on BEHC18 column in negative and positive mode; b: Base peak chromatograms of the mixed standard for **5**, **32**, **42**, **45**, **55**, **86**, and **101**; c: Base peak chromatograms of the mixed standard for **33**, **34**, **47**, **51**, and **77**; d: Base peak chromatograms of the mixed standard for **39**, **52**, **57**, **59**, **68**, **71** and **89**; e: Base peak chromatograms of the mixed standard for **10**, **40**, **53**, **65**, and **73**; f: Base peak chromatograms of the mixed standard for **2**, **7**, **13**, **23**, **29**, **44**, **54**, and **91**; g: Base peak chromatograms of the mixed standard for **4**, **12**, **19**, **43**, **56**, **74** and **102**; h: Base peak chromatograms of the mixed standard for **14**, **16**, **31**, **33**, **61**, **66**, **76**, **85** and **109**; i: Base peak chromatograms of the mixed standard for **1**, **6**, **11**, **35**, **37**, **65**, **73** and **81**; j: Base peak chromatograms of the mixed standard for **38**, **75**, **81**, **83**, **94**, **113** and **114**; k: Base peak chromatograms of the mixed standard for **93**, **94**, **99**, **100**, **102**, **105**, **107**, **111** and **112**
